# An interpretative phenomenological analysis study of perceptions around factors relating to coping amongst young people with Long Covid and mental health difficulties in the UK

**DOI:** 10.1177/20551029261440419

**Published:** 2026-08-06

**Authors:** Madeleine Riley, Georgia Treneman-Evans, Olivia Taylor, Katharine Pike, Nicholas Troop

**Affiliations:** 1 6633 University of Plymouth, UK; 2 1980 University of Bristol, UK

**Keywords:** adolescence, clinical health psychology, health care, children, psychological distress, long covid, covid-19, adolescent, child, mental health

## Abstract

The nature and causes of neuropsychiatric symptoms in children and young people (CYP) with Long Covid are debated in current research. This study explored CYP perceptions of their mental health difficulties in association with Long Covid diagnoses. Nine CYP were interviewed, asking about their experiences of Long Covid and mental health. Data was analysed using Interpretive Phenomenological Analysis. There were two principal findings in this research. (1) Participants related their mental health difficulties to difficulties associated with having Long Covid and wider system pressures. (2) Participants spoke about the impact of stigma in healthcare services delaying access to specialist medical professionals. Findings suggest that healthcare services need to be better informed, and to reduce barriers to access healthcare services. These measures would reduce the pressure on families to fight for services.

## Introduction

### What is Long Covid?

Long Covid or Post-Covid-19 condition is a “multi-systemic illness” (Davis et al., 2023) with persisting physical symptom(s) lasting three or more months after SARS-CoV-2 infection (World Health Organisation, 2022). In the UK 1.9 million people self-reported to have Long Covid in a survey completed in March 2023; 119,000 of these were children and adolescents (Office of National Statistics, 2023).

Children and Young People (CYP) with Long Covid most commonly report symptoms of tiredness, shortness of breath and headaches (Newlands et al., 2023). The condition is reported to have an impact on everyday functioning (Stephenson et al., 2022b) which can be debilitating (Davis et al., 2023). However, there has been a diverse list of symptoms that have been associated with Long Covid in CYP, for example, confusion, ear-ache and hoarse voice (Pinto Pereira et al., 2023). Due to the recency of SARS-CoV-2, first being identified in December 2019 (World Health Organisation, 2022) Long Covid has also only recently been recognised as a condition (Callard and Perego, 2020). It was initially first identified under the name “Long Covid” in May 2020 (Callard and Perego, 2020); therefore, research is still in the early stages to understand and treat this condition.

### Mental health difficulties and diagnoses in people with Long Covid

Symptoms associated with Long Covid in CYP were grouped by Fainardi (Fainardi et al., 2022) into different sub-types, for example gastrointestinal, cardiovascular and neurological/neuropsychiatric. The neurological/neuropsychiatric symptoms associated with Long Covid in CYP were broad, including irritability and mood disturbance, alongside difficulties sleeping, memory loss and headaches. This article will reference neuropsychiatric symptoms as those specifically associated with mental health or mood related disorders, for example low mood, irritability or anxiety. Early indications suggest that CYP with multiple physical symptoms three-months following Covid-19 infection experienced poorer mental health than CYP who did not have Long Covid (Stephenson et al., 2022a).

If Long Covid is to be better understood and treated, it is important to understand the nature and causes of the condition. The causes of neuropsychiatric symptoms of Long Covid in particular are not currently clear. A number of possible causes have been identified by existing research. For example, that neuropsychiatric symptoms might be part of the physical symptom profile (Fainardi et al., 2022). However, another theory suggests that these neuropsychiatric symptoms might relate to stress associated with having Long Covid (Fainardi et al., 2022). The severity and disabling nature of Long Covid, its impact on life goals and enjoyment could also have a significant impact on the mental health of CYP. It is widely understood in the field of psychology that physical illness is a significant stressor (Sharpe and Curran, 2006) and can be disruptive to emotional equilibrium (Carroll et al., 2022). The psychological term “adjustment”, whilst not regularly used with Long Covid populations, notes the importance of considering factors that may impact on the toll and changes associated with having a health condition. For example, having a health condition might cause changes to an individual’s social context (Brennan, 2001), and their appraisal of the consequences of having the condition (Sharpe and Curran, 2006).

Another theory for the causes of higher rates of comorbid mental health difficulties in CYP with Long Covid could be that this is due to misdiagnosis of physical health symptoms as being related to mental health disorders in this population. The malfunctioning of the autonomic nervous system, which occurs with Long Covid can cause symptoms often associated with Anxiety or Depression (Davis et al., 2023; Larkin and Martin, 2017). Furthermore, psychometric tools may lack face validity when used in the assessment and diagnosis of mental health disorders for people with physical health conditions (Grayson et al., 2000). This might impact on people with Long Covid, where symptoms such as fatigue might lead to higher scores on some depression psychometrics (Davis et al., 2023).

Given the scarcity of literature on Long Covid, Myalgic Encephalomyelitis/Chronic Fatigue Syndrome (ME/CFS) is often referred to due to the similar symptoms profile (Davis et al., 2023). ME/CFS is a condition characterised by its persistent and debilitating fatigue, alongside unrefreshing sleep, cognitive difficulties and post-exertional malaise (NICE, 2021). In CYP with ME/CFS there is also a higher percentage of mental health comorbidities (Loades et al., 2020). There is no specific cause determined for the higher level of comorbid mental health diagnoses in this population, however research has hypothesised about factors such as increased loneliness (Hards et al., 2022), and increased anhedonia (Smith et al., 2021). Given the similarities in the symptoms and changes in social circumstances associated with these conditions, for example increased school absences (MacLean et al., 2023); research findings about loneliness and anhedonia, which are noted in the research about CYP with ME/CFS, might also impact CYP with Long Covid.

### The impact of wider social and medical systems on people with Long Covid

CYP with Long Covid reported more frequent experiences of loneliness than the general population of CYP (McOwat et al., 2023). However, current research does not address the causes or exacerbatory factors of loneliness for this population. In adults with Long Covid, isolation is noted to be exacerbated by the reduction in social activities and unemployment or sick leave (Schmachtenberg et al., 2023). Similar factors might impact CYP. For example, a qualitative study notes that participants found absences from school stressful and isolating (MacLean et al., 2023).

Research so far has suggested that disbelief by medical professionals, or incorrect beliefs that the condition is a mental health disorder, can delay treatment and cause stress for CYP with Long Covid (Torres et al., 2024). It is noted in Long Covid CYP literature that it is important for healthcare professionals to provide support and validate CYPs’ experience to “minimise adverse educational, social and mental health sequelae” (MacLean et al., 2023). However, one factor which has been documented in the adult Long Covid literature, is the impact of what is sometimes termed “medical gaslighting” (Owen et al., 2023), where medical professionals invalidate or ignore concerns raised by patients (Godman, 2024). This is an important factor in understanding extra stressors for people with Long Covid, and therefore factors which may impact on mental health and the likelihood of seeking help for mental health difficulties. This research seeks to understand whether medical gaslighting may also impact on CYP with Long Covid, and how this may impact on CYPs’ mental health difficulties and access to healthcare.

### Treatment of Long Covid neuropsychiatric symptoms

Due to the novelty of Long Covid and current limits to the evidence base, currently no specific treatments are recommended for CYP with Long Covid (Garai et al., 2022). However, in a rapid review issued in December 2020, and last updated in January 2024, the National Institute of Health and Care Excellence (NICE) noted the importance of providing resources and support to enable self-management, including careful self-pacing of exercise in their guidance for adults with long-term symptoms following Covid-19 infection (NICE, 2020).

Despite the limitations to the evidence-base at present, psychologists highlight the importance of psychological support for people with Long Covid for alleviation and improving management of physical health symptoms such as brain fog, insomnia and fatigue (Schreiber, 2021). Furthermore, a qualitative study collected the experiences of therapists working with people with Long Covid (*n* = 16; Burton-Fisher and Gordon, 2024). Therapists note the value of using interventions that consider the psychological concept of hope (Snyder, 2002), which is reported to support people with Long Covid to develop agency and motivation to work towards recovery goals (Burton-Fisher and Gordon, 2024). However, this study does not confirm the age groups the therapists are working with. Therefore, it is difficult to ascertain the transferability of these results to CYP with Long Covid.

Systemic formulation has been used in ME/CFS literature. A systemic approach might consider mental health difficulties as the product of relational dynamics or a symptom of a wider problem in the system, rather than an individual difficulty (Carr, 2012: p. 69). This approach may be helpful for mapping out the different levels of support or indeed interpersonal difficulties a CYP might face when they have a complex health need (Brigden et al., 2020; Lewis, 2022). For example, Lewis (2022) recommends that improvement to the communication pathways between healthcare services and educational systems may improve the psychological wellbeing of the CYP (Lewis, 2022). The similarities in interpersonal and organisational difficulty for CYP with ME/CFS and Long Covid, may suggest this theory can also be helpfully applied for CYP with Long Covid.

### Specialist service provision

The National Health Service (NHS) developed specialist post-covid services in England between 2021 and 2022 (NHS England, 2022). Services are expected to operate using the recommended multi-disciplinary model, including access to specialist mental health professionals or psychologists, alongside medical and functional assessment (NICE, 2020).

The extent and longevity of funding for specialist Long Covid services remains uncertain, with current funding extending only until 2025 (Alexandri et al., 2024). Meanwhile, some services have closed already, such as West Essex (Essex Partnership University NHS Trust, 2023). A reduction in the number of specialist Long Covid services in England could have a negative impact on the wellbeing of people with Long Covid, given these services may offer a space where people with Long Covid feel understood and their condition is legitimised and validated (Sunkersing et al., 2024).

### Study aim

On the basis of the literature reviewed, this study aims to explore CYP perceptions of their mental health difficulties in association with Long Covid diagnoses. Furthermore, to explore CYPs’ perspectives of the efficacy of psychological interventions. CYP were therefore asked about wider stressors and difficulties related to having Long Covid, and the impact these systemic factors had on their mental health and experience of their condition.

### First author researcher positionality

I self-identify as white and female, with an unseen disability. In my childhood I had experiences of being disbelieved by healthcare professionals when seeking diagnosis of my disability. I have spent time meeting with people with Long Covid, who often share their view that it is a physical health condition where a physical cause is yet to be identified, and yet they speak about being told it is a mental health condition by healthcare professionals. I feel it is important to highlight and assume that Long Covid is a physical condition with a physical cause, as this is what is reported by many experts by experience.

## Methods

### Participants and recruitment

This study is one arm of the wider LoCATE study. The LoCATE study was undertaken to explore CYPs’ experiences of accessing a specialist Long Covid service. Participants for both arms of the LoCATE study were recruited from a Specialist NHS Service in the South-West of England. Participants were recruited by staff from the service who spoke to CYP when they were accessing services for assessment or treatment sessions. As a Long Covid diagnosis was required to access treatment within this service, this ensured all participants in the present study had a Long Covid diagnosis.

To be included in the study, participants had to be aged between 11 and 17 years old, have a diagnosis of Long Covid, and be currently receiving support from the service. Furthermore, for the mental health specific arm of the LoCATE trial, participants were screened and were only included in the study if they met at least one of the following criteria:

(1) At least one above threshold score on the Revised Children’s Anxiety and Depression Scale (RCADS; Chorpita et al., 2000).
(2) If they had seen a psychologist or other mental health professional as part of their treatment.

These screening measures aimed to focus on CYP with Long Covid who had experienced mental health difficulties and either received psychological support or developed ways of managing their mental health difficulties outside of service support. Focusing on a group of participants who had experienced mental health difficulties and/or accessed mental health support improved the homogeneity of the participant sample.

Potential participants were excluded from this study in the following circumstances:

(1) They were below 11 or above 17 years old.
(2) Anyone who had not been seen by local specialist services following a diagnosis of Long Covid was unable to participate in this study. This ensured a ratified Long Covid diagnosis had been given to all those who participated.
(3) Any participants who had either not accessed mental health support in connection to their Long Covid or had not scored above threshold levels on the RCADS.
(4) Participants who did not speak English, as interviews were conducted in English.

Twenty-four CYP agreed to be contacted as part of the wider LoCATE study, where they could be invited to two interviews, and alongside this asked to complete a battery of outcome measures. They were sent outcome measures by email, including the RCADS. Participants were contacted by email to invite them to attend the interviews and, if they consented, they were sent information about the interview and purpose of the study for themselves and parents/guardians.

### Interview

Due to the novelty and limitation in the evidence base in this area of study, an exploratory focus in the collection and analysis of the data enabled flexibility to focus on the factors participants felt were most important to discuss. Semi-structured interviews enabled participants to share information otherwise not asked about, but relevant to the topic.

The interviews lasted approximately 1 h. Participants were offered face-to-face or online interviews. One was carried out face-to-face in a clinical setting and 8 were online. The interview questions are available in Appendix A. They were open-ended, broaching subjects such as participants’ experiences of living with Long Covid and their perceptions of their mental health and mood. All interviews were conducted by one researcher, a Trainee Clinical Psychologist employed by the NHS. Participants were asked at the end of the interview if there were any topics that they felt they had not been asked about that they wanted to share further information on. Participants were given the option for parents or carers to attend the interview with them for support. Each participant received a £20 voucher as a thank you for participating in the interview.

### Data analysis

Interpretive Phenomenological Analysis (IPA; Smith and Nizza, 2021) is helpful for providing a rich and in-depth account of the lived experience of the participants. In updating the process of analysing data in IPA, Smith and colleagues (Smith et al., 2022; Smith and Nizza, 2021) introduce new terminology, Rather than *emergent themes*, *superordinate themes*, and *master themes*, analysis refers to *experiential statements*, *Personal Experiential Themes* (PETS), and *Group Experiential Themes* (GETs). PETs enable consideration of the variation of the symptoms and experiences of each participant, and an understanding of how each participant’s unique circumstances might change their interpretation of their own narratives. This was particularly important to consider due to the variation in symptoms reported associated with Long Covid, and capturing unique perspectives from the social and family circumstances and other diverse factors. The individual analysis component of IPA was chosen as it enables each participants’ experience to be focused on independently participant experiences are then considered as a group, through Group Experiential Themes (GETs), identifying patterns of convergence and divergence.

Each script was transcribed by the researcher in order to gain familiarity with the content. It was then read through at least twice, with notations (1) to identify experiential statements, (2) to acknowledge factors appropriate for bracketing, for example, the thoughts and feelings more personal to the researcher. Bracketing allowed the researcher to “question their assumptions” (Barrett-Rodger et al., 2022), where these assumptions might otherwise bias the research process. The experiential statements of each script were exported to their own Miro document (Miro©, 2024). Miro is a concept mapping software enabling quotes to be sorted and clustered. Once clusters were defined and PETs emerged, the researcher moved on to the next script to complete the same process. The researcher brought anonymised transcripts to a peer consultation group containing three other researchers. These spaces supported the development of alternative hypotheses, and managed some of the biases that might otherwise have been of concern due to the researcher’s personal experiences. These hypotheses were used, in conjunction with the researcher’s experience-informed interpretation, to create a broader interpretation of the meaning of participants’ experiences. Furthermore, presuppositional interviewing (Barrett-Rodger et al., 2022), research supervision and bracketing were also used to note where personal circumstances might impact on the analysis of the data. The PETs were then merged through another Miro workspace to be re-clustered to establish GETs, approximately 50% of this was completed in research supervision sessions to support bracketing.

To compare each participant’s experiential statement, and the “convergence and divergence” of their experiences, the researcher collated the PETs into GETs (Hefferon and Gil-Rodriquez, 2011). This process is helpful when reporting on the variation of the different experiences the participants shared because there can be no expectation that any two participants will share the same perspective on a given experience. GETs purposefully demonstrate the interplay between the different participants’ perspectives and acknowledge where the researcher’s interpretation has also been taken into account when creating a narrative and a deeper understanding of the PETs. Acknowledging that the researcher’s interpretation was also considered, alongside the participants’ individual experiential statements, enables a broader understanding of a theme and in turn enables the reader to create their own interpretations of the research presented (Eatough and Smith, 2006).

### Ethical considerations

The research received ethical approval by the Health Research Authority (HRA) and approval from the University of Plymouth Ethics Committee.

Before the interview, participants were offered different adjustments. For example: a range of options for interview location, including online, at home or in their local clinic. Participants were offered breaks, multiple shorter sessions, and date and time flexibility to reduce the risk of *post-exertional malaise*. The participants were aware they could leave the interview at any time. Some participants requested parents attend the interview, often to support the participant with their symptoms. A protocol was in place to manage disclosures of risk-related information if they had occurred.

Consent was taken verbally using a voice recorder. Consent was asked from each CYP, and also obtained from parents for participants under the age of 16. Where CYP requested their parents stay for the interview, consent was also taken from parents for their comments to be included in the analysis and research.

## Results

Of the 24 participants who agreed to be contacted as part of the wider LoCATE study, five were screened out of this arm of the study due to the inclusion and exclusion criteria. As such, 19 CYP from the wider LoCATE study were invited to interview. Of the 19, nine participants attended semi-structured interviews and completed RCADS questionnaires. Of the other participants, the majority did not respond to contact attempts by email and several participants were too unwell to attend the interview. Eight out of nine participants who were interviewed also consented to RCADS questionnaire information being published as part of the research study. All participants were female and aged between 13 and 17 years old at the time of the interview. All participants described themselves as white, and eight of nine as English, Welsh, Scottish, Northern Irish or British, with one describing themselves as white, other.

Of the nine participants, parents of one participant did not consent (NC) to the participants’ RCADS scores being published, these scores have therefore not been included in Table 1. Of the other eight, six scored within the clinically severe category for depression and one within the clinically severe category for anxiety. All but one participant disclosed being seen by a mental health professional either privately or with the specialist service.

**Table 1.** Participant demographics and clinical characteristics.

Participant number	Age	Ethnicity	Depression	Anxiety	Additional severe sub-scale scores	MH professional involved in treatment/support	Parent attended interview
1	15	White UK	Severe	Severe	Generalised anxiety disorder, separation anxiety, social anxiety	Yes	Yes
2	14	White UK	Severe	Mild	Separation anxiety	Yes	Yes
3	16	White other	Mild	Mild		Yes	No
4	13	White UK	Severe	Mild		Yes	Yes
5	17	White UK	Mild	Mild		Yes	No
6	15	White UK	Severe	Mild	Separation anxiety	Yes	No
7	17	White UK	Severe	Borderline	Panic disorder	Yes	No
8	16	White UK	Severe	Mild		Yes	No
9	14	White UK	NC	NC	NC	No	Part

### GETs

Participants referred to a number of different aspects associated with their experiences of having Long Covid. These could be outlined through the themes and sub-themes below (Table 2).

**Table 2.** Themes and sub-themes.

GETs	Sub-themes
GET1: Persistence and severity of symptoms	1. Everyday relentlessness of symptoms
	2. Disabling nature of symptoms
	3. Missing out
GET 2: Support networks and isolation	1. Family support
	2. Peer misunderstanding and disbelief
	3. Peers with long covid
	4. Supportive friendships
GET 3: Experiences of accessing healthcare services	1. It’s all in your head
	2. Not being believed
	3. Specialist interventions and support
GET 4: Educational access and attainment	1. Access to education
	2. Impact on attainment
GET 5: Impact on mental health and identity	1. I wasn’t myself
	2. Mental health and adjustments difficulties

### GET 1: Persistence and severity of Long Covid symptoms

The symptoms participants described varied significantly between participants. Symptoms were often described as relentless and disabling.

### Sub-theme 1: Everyday relentlessness of symptoms

Participants spoke about the everyday nature of Long Covid symptoms, with a sense of symptoms being constant or relentless. *“I always have a constant headache” (Participant 1)*. Participants spoke about it becoming their “*normal*” and feeling they *“kind of just get used to it” (Participant 4)*. This may be due to participants often having had Long Covid for two or 3 years prior to the interview.

### Sub-theme 2: Disabling nature of symptoms

Participants spoke about how disabling their symptoms were in stopping them from doing tasks that prior to having Long Covid they would have considered *“simple” (Participant 1); “washing my hair really like wears me out” (Participant 1)*. Participants used examples that illustrated the extremity of their disability: *“I physically can’t pick up the cup with heavy water in.” (Participant 7)*.

### Sub-theme 3: Missing out

Finally, participants spoke about their symptoms causing them to miss out on enjoyable activities. Participants often referenced their age and stage of development, and how this impacted on the importance of engaging in social activities.

“Especially when you’re like 17 and you’re just like getting to the level where people want to do stuff,[…] I physically and emotionally and mentally can’t do any of that” (Participant 7).

Participants found it difficult, particularly on social media, when comparing what their peers were doing with their own limited activities due to their Long Covid diagnosis.

“If you open up Instagram and see all of your classmates, a big one for people my age is prom. I know some people with Long Covid have been able to go to prom, but I wasn’t able to go to prom” (Participant 6).

On the other hand, some participants chose at times to not miss out on doing things. They noted that they would experience a worsening of physical symptoms as a result.

“We went [to the music concert] and it was amazing… six days after, I was like unable to walk, unable to get out of bed… because you can’t just put your life on hold just to stop that” (Participant 7).

### GET 2: Support networks and isolation

Participants spoke about the impact and importance of their support networks. Often prior to any prompts asking about support, participants spoke about where either their support networks had made things more hopeful, left them feeling reassured and supported; or alternatively led to feelings of isolation, feeling left out or misunderstood. This therefore often seemed to be a priority and a key aspect of participants’ experiences.

### Sub-theme 1: Family support

Some participants requested that their parent attend some or all of the interview. Participants were asked about their support systems, and whether their family were supportive, and the attendance of a parent is likely to have an impact on the answer the participant gave. However, the presence and connection seen during some interviews between participants and their parents demonstrated the key role parents provided in supporting some participants.

For many participants, they primarily spoke about their mothers who often took a role in researching Long Covid and advocating for the participant to be able to access appropriate services: *“mum was very persistent in requesting fatigue clinic” (Participant 8)*. Participants also described the importance of family in alleviating feelings of isolation:

“My sister would come in my room […] I would do her hair or she would do my make-up or something. It was that whole thing of keep that interaction. Because that’s what I felt helped me before I started talking to someone” (Participant 7).

Overall, the closeness and supportiveness of family relationships was noted by participants. *“My mum was my closest support throughout the whole thing” (Participant 9)*.

For participants where they felt less able to open up to family about the impact of Long Covid on their mental health, this might impact on them asking for help from services.

“I don't know where to go for [mental health support]. And I don’t really want to, have to like, go and ask my mum for it. Because of course she’d get it for me, but then she'd be very, very worried” (Participant 8).

For this participant, it is unknown how the mother or wider family perceive mental health difficulties. However, such worry and concern from the mother might indicate incorrect beliefs around mental health difficulties being abnormal, rather than a common aspect of Long Covid symptoms.

### Sub-theme 2: Peer misunderstanding and disbelief

Participants spoke about not wanting to be asked questions about Long Covid by peers or friends due to finding it difficult to answer questions.

“It’s so hard to explain what Long Covid is because […] there’s so many symptoms. […] You can’t just say I’m really tired, that’s what Long Covid is because it’s not that. And if you do say that, then people don’t take you seriously” (Participant 2).

Participants spoke about feeling that classmates and friends didn’t understand the realities of having Long Covid. *“They were saying like, take one for the team, get Covid so we can all go home” (Participant 7)*. Participants described this as *“isolating” (Participant 3)* or *“frustrating” (Participant 8)*.

Participants also spoke about the impact on feeling disbelieved or misunderstood by peers: *“people don't believe me. I start not believing myself […] Is it just something in my head? It’s terrifying” (Participant 2)*.

### Sub-theme 3: Peers with Long Covid

Many participants spoke about being the *“only one” (Participant 3)* with Long Covid. This experience seemed to further exacerbate feelings of *“loneliness” (Participant 8)*. However, for those who did have opportunities to speak with other people with Long Covid this was often seen as *“helpful” (Participant 1)*. Participants in particular spoke about online groups set up through the LoCATE study for the purposes of involvement and engagement with the research project.

“They think there are no people at my school with ME or Long Covid. And that was quite a shock to both me and mum. But yeah, it was really nice to see that other people have and knew what I was going through” (Participant 2).

### Sub-theme 4: Supportive friendships

Some participants found ways of managing feelings of isolation, depending on whether they had supportive friendships. Good friendships often were noted by participants as being accommodating of the participants’ disabilities. For example, choosing inclusive activities such as going to the cinema rather than going for a walk, or noticing signs of illness in the participant. *“My other friend always double checks out if I’m like tired. If you want to stop what we’re doing or something” (Participant 1)*.

Some participants also spoke about Long Covid being positive in that it demonstrated the strengths of their friendships. *“There’s that whole thing of like, you learn who the people are that actually want to be a part of your life” (Participant 7)*.

### GET 3: The mental health implications of difficulty accessing healthcare services

Overall participants spoke positively about their experiences accessing specialist healthcare services for Long Covid. However, they often spoke about their difficulty in initially accessing primary or acute services for diagnosis. Participants spoke about not being believed or misdiagnosis with mental health conditions.

### Sub-theme 1: It’s all in your head

Participants spoke about the difficulties they faced in diagnosis of Long Covid and gaining access to Long Covid specialist services. Some participants prior to receiving a Long Covid diagnosis, were misdiagnosed with a mental health condition: *“In my old doctors, they used to say it was all in my head” (Participant 7)*. Another participant spoke about being inappropriately referred for their Long Covid symptoms to receive mental health support, where they were put in a group to go on therapeutic walks (which would be contraindicated for Long Covid management due to the risk of *post-exertional malaise)*. Their parent noted supporting their child to advocate for access to Long Covid service support:

“‘Yeah. It’s not that my mental health is making me ill’ [the participant] said, ‘I’m down because I can't do anything’,” (Parent of Participant 1).

Another participant noted experiencing extreme physical symptoms due to Long Covid, and accessing A&E: *“I was keeled over in agony with my stomach […] and they told me about, you know, the mind gut connection and […] was I stressed recently? And then I had a test and [the test result] was 14 times over the limit” (Participant 6)*.

### Sub-theme 2: Not being believed

Alternatively, some participants had difficulty receiving a diagnosis due to not being believed. They noted the difficulties of trying to access support and feeling professionals were not listening or believing them.

“For the first session [with an NHS Paediatrician], he wouldn’t believe it was Long Covid. The second session we, he forgot who we are and we had to re-convince him it was Long Covid” (Participant 8).

Participants spoke about how this may impact on wanting to access healthcare services in the future.

“If you’re fatigued to the point where you can’t get out of bed or you're keeled over in pain and then you walk into a doctor’s office, and you’re being told you’re faking it. Well, why would you ever want to go back to the doctors again?” (Participant 6).

### Sub-theme 3: Specialist psychological interventions

Due to the focus of this research on mental health impact and support, participants were asked specifically about whether they found any mental health support or interventions helpful. The participants in this study seemed to outline two forms of support offered by the NHS specialist service. However, it was difficult to differentiate which professionals were offering these different models, as participants often didn’t know the role or names of the professionals they were working with.

Participants spoke about the specialist clinicians often working in a consultative or advisory capacity, developing *“coping strategies” (Participant 8)* to manage fatigue or reduce gastro-intestinal symptoms. Whilst participants spoke about the value of the consultative, psycho-educational approach, they also highlighted that they felt they would have benefited from a wider therapeutic approach where emotions and adjustment to their condition were talked about.

“We brushed over mental health in the clinic thing. But I think just some extra like support with that would have just made me feel better” (Participant 5).

On the other hand, Participant 7 spoke about having a greater number of sessions where they built a trusting *“bond”* with the clinician, *“working through things”* unconnected with current Long Covid symptoms. This participant noted that they had a history of bullying and was a young carer; these factors may have impacted on their needs for more in-depth psychological support.

### GET 4: Educational access and attainment

Participants spoke about the impact Long Covid had on their access to education, and in turn the impact they perceived this might have on future attainment in exams or careers. Often their concerns about attainment were dependent on their age at the time of the interview, which could impact on the pressures they experienced when in school.

### Sub-theme 1: Access to education

All participants spoke in depth about education. For most participants they had missed a substantial amount of school and had felt worried about the impact their absence would have on their education. This was for some also exacerbated by the cognitive symptoms associated with Long Covid.

“I was forgetting things and I wasn’t retaining information for like exams and stuff which like hindered my ability to perform at school” (Participant 3).

A key factor associated with this was the understanding and level of support that could be offered by schools. Participants seemed to have a range of different methods of accessing education. For some this was attending no school and then gradually building up attendance when recovering. Other schools were seen as *“really good” (Participant 8)* for allowing online access to usual classes and calls with teacher. This is in contrast to other participants who were unable to access even basic teaching in some subjects, or received hospital/alternative educational provision (PRU), which was described as limited, often only providing teaching in English and Maths *(Participants 1 and 6)*;*“six subjects I had to teach myself completely independently with no help” (Participant 6)*. For those who felt unsupported with their learning, they spoke about having to *“power on despite”* poor support *(Participant 6)*.

### Sub-theme 2: Impact on attainment

Participants spoke about how the usual worries experienced by teenagers around exams were exacerbated by school absence and having to manage their Long Covid symptoms while sitting exams.

“All of those worries that you have in the future like GCSEs, what I’m doing after school, just like generally, it was really scary” (Participant 2).

Participants completing GCSE or A-Level study spoke about worrying that poor access to education could impact on their attainment in exams or their future careers. In particular, those who were 17 years old spoke about the impact their Long Covid symptoms could have on applications to university or career prospects.

“I wanted to be a pilot in the RAF, it was my dream since I was 4 years old[…] and now, there’s not a 100% that I would get back to full physicality, which means, there is no way that I will get into the RAF” (Participant 7).

“I’m doing like early application for university and I feel like now I’m kind of realising how much I haven’t done because of my fatigue. […] Like I would have read more and I would have done this other class, but I can’t […]. Actually, the reason it’s not going the way I want it to is because of Long Covid” (Participant 5).

### GET 5: Impact on mental health and identity

Participants noted the impact Long Covid had on who they were and how they were feeling. Some spoke about feeling like a different person, while others spoke about severe low mood and feeling at rock bottom. Some participants were able to explain how this often linked with their physical symptoms and inability to do the things they used to do or would find helpful for their mental health. Furthermore, many participants spoke about an improvement in their mood as they started recovering.

### Sub-theme 1: I wasn’t myself

Participants spoke about how Long Covid led to an impact on their identity or sense of self. This often correlated with them being unable to do activities that were important to them. *“You’re not the same person that you used to be because you can’t do these things” (Participant 7)*. One participant who lost the use of her legs as a result of Long Covid said *“I think that [losing my legs] took a really big toll on me. […] I couldn’t go into school and do full days. That's not me at all” (Participant 9)*.

Some participants also referred to the impact on their identity of adjusting to having a disability and wanting to be normal.

“Having to use a lift, just having all this, all these teachers constantly checking in on you. […] I don’1t want that special treatment. I just want to be a normal girl” (Participant 9).

Furthermore, participants spoke about becoming *“quieter” (Participant 8)* due to Long Covid. *“I just was a bit more withdrawn because I felt like I wasn’t, I didn’t have anything of importance to say*” *(Participant 3)*.

### Sub-theme 2: Mental health and adjustment difficulties

Some participants seemed reluctant to share information relating to mental health difficulties. It was recognised how difficult this topic could be to speak about in an interview, especially for participants who have experienced previous stigma around their mental health.

“Parent: You get upset. Then you get down about it all don’t you?

Participant: No.

Parent: No? Well, you sometimes say you do then” (Participant 4 & their parent).

On the other hand, some participants spoke about feelings of low mood associated with their Long Covid. Participants seemed to associate these experiences with the worsening of their physical symptoms. *“Like I was basically in a dark hole and my illness got to rock bottom*. *[…] because it got so bad I could hardly move” (Participant 7)*. This could occur alongside feelings of hopelessness and desperation, especially when participants had experienced symptoms for a longer timeframe without feeling they were recovering. *“It was a couple months later and I still wasn’t better” (Participant 1)*.

Participants noted that Long Covid symptoms were preventing them from accessing activities that they would usually do to improve their mental health. *“The stuff I used to do to help my mental health or to help me in general would be like, going for a walk, going to do a workout” (Participant 7)*. Participants often noted that mental health difficulties *“improved”* as they recovered *(Participant 5)*.

## Discussion

This study explored the experiences of CYP with Long Covid and mental health difficulties, focusing on the difficulties they experienced and how they coped. This included exploration of wider, systemic factors impacting on CYP with Long Covid, such as their experiences of accessing support from Long Covid services. This study hoped to explore the usefulness of psychological intervention for CYP with Long Covid. This was undertaken through interviews with CYP with Long Covid, and the use of IPA to establish GETs. Two principal findings were established when synthesising these results and reviewing the existing literature.

## Findings

### Finding one: Participants related their mental health difficulties to difficulties associated with having Long Covid and wider system pressures

Participants spoke about how the five GETs identified in this study, alongside additional themes that arose from the literature, could have a significant negative impact on their mental health and wellbeing. For example, participants reported in this study the disabling and persisting symptoms they experienced due to having Long Covid. This is consistent with the disabling nature highlighted in other studies of Long Covid (Davis et al., 2023), particularly symptoms of tiredness, shortness of breath and headaches (Newlands et al., 2023).

Stigma or poor support from peers was noted to be particularly difficult by participants in this study. Furthermore, CYP with Long Covid reported more frequent experiences of loneliness than the general population (McOwat et al., 2023). The impact of Long Covid on education and attainment was also noted as a stressor in this study. MacLean et al. (2023) also reported that CYP found absences from school stressful and isolating.

One area that has been less reported in previous research has been those factors which supported CYP in coping with Long Covid. Participants in this study reported protective factor*s* and factors which they felt reduced mental health difficulties. Wider system supports were helpful in participants managing their condition, for example: supportive access to education, emotionally and practically supportive family, peers who were accepting and willing to adjust their activities to improve accessibility, and medical professionals who believed them. In particular, participants spoke about how the support they received from family reduced experiences of social isolation and low mood. Equally, some participants spoke about the positives they gained from knowing which peers were supportive of them. Whilst no research was found relating psychological systemic theory to Long Covid, similar themes emerge in the literature around CYP with ME/CFS (Brigden et al., 2020; Lewis, 2022). This research speaks to the importance of including wider support structures (for example: schools, friends and family) in supporting CYP with their physical health and other difficulties associated to the condition.

Furthermore, where these wider systemic support factors are not present, it might lead to a greater level of distress and maladaptive adjustment to Long Covid diagnosis and treatment. This connects to the wider literature on adjustment theory, which considers factors such as the wider social context (Brennan, 2001), alongside the appraisal of consequences of the health condition, for example, likelihood to get into university (Sharpe and Curran, 2006) as factors that will impact on the ability to successfully adjust to their condition. Application of similar systemic theories (as used in the CFS/ME literature) might assist with knowing how to offer interventions to the systems currently impacting on CYP with Long Covid.

### Finding two: Participants spoke about the impact of stigma in healthcare services delaying access to the correct medical professionals and impacting upon willingness to seek help from services

Participants spoke about seeking help from healthcare services, and being given mental health diagnoses that they felt did not fit their experience of their condition. This seemed to be a wider experience noted in the research about Long Covid (Owen et al., 2023; Torres et al., 2024).

One consideration is whether mental health symptoms are pathologised. Factors such as societal beliefs, misinformation in healthcare professionals, and incorrect use of psychometrics (Davis et al., 2023; Grayson et al., 2000) might be important to understand the distress and difficulties noted by participants in this study. It is important to note that some participants were reluctant to speak about mental health difficulties in the study or spoke about difficulties in reaching out for support from healthcare services due to the risk of mental health diagnosis.

#### Findings synthesis

**Figure 1.**
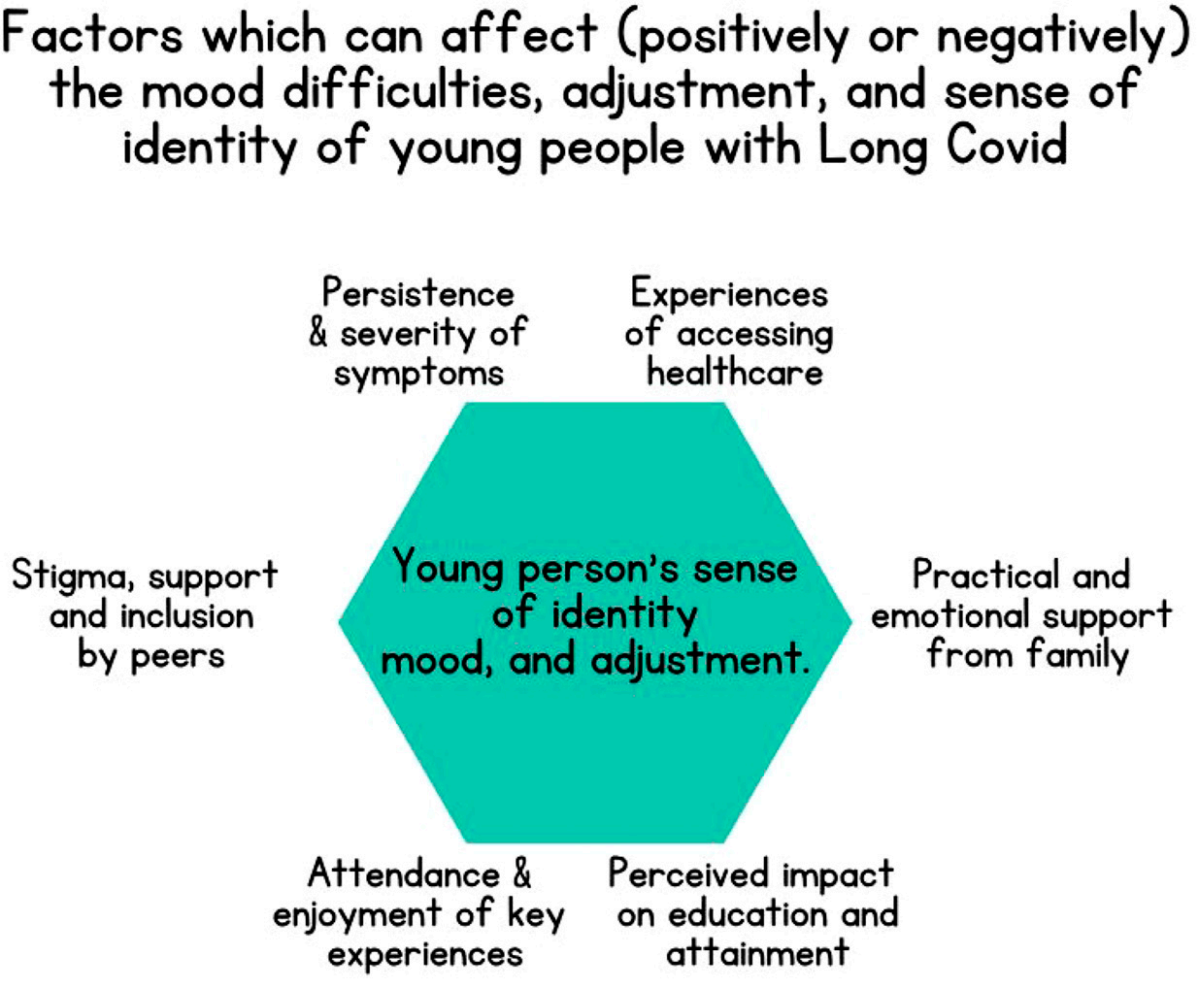
Factors which can affect (positively or negatively) the mood difficulties, adjustment, and sense of identity of young people with Long Covid.

Participants in this study noted multiple factors that impacted on their mood, identity and overall wellbeing. These factors are summarised below in Figure 1. Figure 1 synthesises the key literature findings alongside the results of this research to consider the important factors currently known, which may be helpful in the assessment and psychological support of CYP with Long Covid and mental health difficulties. As such “attendance and enjoyment of key experiences”, “stigma or support from peers”, and “practical and emotional support from family” in Figure 1 do not map directly onto the results of this study, but are important study sub-themes or are prominent points from the literature review.

The sub-themes for peer related support or stigma have been grouped, however distinguished from family-related support, as both factors were important for wellbeing, but sometimes had different roles in supporting the participants. Attendance and enjoyment of key experiences closely maps to the sub-theme of missing out. However, this factor also highlights the importance of attending enjoyable events for improving the mood of participants as a potential protective factor for mental health.

#### Strengths and limitations

By choosing an IPA methodology this study is limited in the transferability of the findings (Smith et al., 2022). However, the similarity in gender, age group, and diagnosis enabled a good similarity in experiences throughout the sample. This is used in an IPA methodology to improve insight into a particular experience (Smith and Nizza, 2021). One limitation in this study may be the screening measures used, which purposively selected for CYP that were more likely to have experience of neuropsychiatric symptoms or of needing support from mental health professionals. While the RCADs is commonly used in the assessment of CYP with Long Covid and other physical health conditions, it has been noted in this study that it may lack face validity for this population.

Another consequence of the screening process is that participants recruited for this study are not representative of the general population who can have Long Covid. Participants were all CYP, between the ages of 13 and 17. All participants self-identified as female and white. This is likely to have impacted on the results reported, especially when noting factors such as being believed by peers and medical professionals. Gender was not purposively selected for and the number of female participants might be due to a higher prevalence of female CYP reported to have Long Covid (Kikkenborg Berg et al., 2022; Stephenson et al., 2022). It is likely some CYP with Long Covid will have different experiences of discrimination around their Long Covid, due to intersectionality around characteristics such as gender, race, ethnicity, culture, and socio-economic status.

One factor that is likely to have impacted on the interview and interpretive process is the lived experience of the researcher. The researcher’s experiences of disability and difficulties in accessing education and healthcare, at a similar age to that of the participants, can be viewed as both a strength and limitation of this study. The researcher’s personal experiences may have enabled her to pick up on underlying nuances in the interviews, and support participants to communicate complex or difficult experiences. However, these experiences may also have increased risk of bias in the researcher’s choice to probe further on aspects of interview content. The researcher may have also been more likely to pick up on themes that connected with their own lived experiences. These biases have been mitigated through the use of supervision, consultation and bracketing. As noted in Finding two, some participants felt uncomfortable speaking about mental health diagnosis. One limitation of this study is that the interviewer is a Trainee Clinical Psychologist, employed by the NHS, and recruitment took place through NHS staff. These factors may have impacted in some cases on how comfortable participants felt speaking about their mental health and service access difficulties.

A potential methodological limitation of this study was the inclusion of parents in attending some of the interviews. This was an ethical decision and was the young person’s choice. Nevertheless, this may have impacted on what CYP felt comfortable sharing in interviews. Conversely, others spoke about feeling more comfortable talking about their experiences with their parent present. In addition, the researcher was vigilant to ensure that CYPs’ voices were prioritised.

#### Implications

The findings of this study show that the primary emphasis needs to be recommending changes to healthcare professional conduct to provide an affirmative space for CYP with Long Covid, and to support referral and identification of Long Covid across healthcare services. This study identified a model that could be used to support assessment of mental health difficulties and risk factors for CYP with Long Covid.

Furthermore, this study highlights the helpfulness of specialist Long Covid support for CYPs’ mental health. This implication is important especially in light of the uncertainty around future funding for Long Covid specialist service provision (Alexandri et al., 2024).

This study also brings to light the “Adjustment” model used in wider Clinical and Health Psychology. We note that this model does not appear in the literature we have seen on Long Covid in CYP. Further consideration of this theory and how it could be related to CYP with Long Covid would be helpful in future research. This study also highlights the use of systemic psychological theory in framing and understanding the needs of CYP with complex health conditions. It recommends that similar uses might be helpful for future Long Covid research and clinical practice.

We also identified wider implications in terms of the need for accessible and equal schooling provisions for CYP with Long Covid. These may also have implications for wider educational and disability-related policies. However, it was noted that whilst the primary findings indicated the need for improving access to services, these findings may also have implications for the need for greater resource provision to be offered to the public in order for families and carers to advocate for CYP with Long Covid when having difficulty accessing services and education.

The findings of this study include implications for further research. Due to the limitations in this study, we recommend further research to understand how diversity in characteristics such as gender, race, ethnicity and socio-economic status may impact on the frequency and severity of stigma for CYP with Long Covid, particularly from peers and healthcare services. Furthermore, it is recommended that a study to determine how commonplace factors such as medical gaslighting and difficulties in accessing education are for CYP with Long Covid, using a larger population and quantitative methodology. Further research could also be conducted into understanding the long-term consequences of Long Covid and the systemic stressors associated with it for mental health, education and employment.

Further research on the use of the RCADS with CYP with Long Covid would be helpful for understanding whether there is a need to improve its validity or to use alternative psychometrics in future research to better understand how mental health is affected by Long Covid symptoms. This could also have positive implications for recommending the most fit for purpose psychometric tools for Long Covid specialist services.

## Conclusion

This study has observed multiple factors which impact on the mental health difficulties faced by CYP with Long Covid. Some of these factors have a negative effect, and some positive. Participants also reported difficulties with stigma and over-pathologisation of their mental health related symptoms, which are corroborated with current literature. Participants described experiencing stigma related to their mental health diagnoses or difficulties. They also described how mental health diagnoses actually created barriers to accessing appropriate healthcare services.

## Data Availability

Due to the nature of the research, and for ethical and confidentiality reasons supporting data is not available.*

## Appendix

### Appendix 1

Version: 1

Date: 10/03/2023.

#### Topic guide for semi-structured interviews focused on the perceived mental health needs and psychological treatment experiences of adolescents with Long Covid

Tell me about your experience of living with Long Covid?

- Prompt: How has it affected you?

Has having Long Covid had an impact on your mental health/overall mood? -

- Prompt: Could you tell me more about how it has affected you?
- Prompt: How has your mental health changed since you started meeting with the service?

What was it like to meet with the clinical team?

- Prompt: How did you feel about being referred to a psychologist? (Note to interviewer: explore experience of stigma if discussed)
- Prompt: Is there anything that the psychologist could have done differently that could have helped you?
- Prompt: From meeting with the psychologist, is there anything you have taken away with you?

How have you found it to fill out the forms/answer the questions from the service? (E.g. RCADS).

- Prompt: Sometimes they can feel really tiring, or they can help us realise things that are wrong that we might not otherwise notice. What do you think?

What other support have you had for your mental health outside of the service? E.g. mental health services, professionals, parents, carers, friends, online.

- Prompt: What was your experience of the support you had?
- Prompt: Was it helpful or unhelpful?

What support would you have wanted? E.g. physical health, mental health, professionals, friends, family.

- Prompt: What support do you feel you still need?
- Prompt: What support would you like in the future?

What advice would you give someone else who was coming into the service with your difficulties?

- Prompt: Was there anything that worked for you while you were waiting to be seen by the service?

Is there anything else that you would like to discuss?
